# Supplementary material for: Chromothripsis during telomere crisis is independent of NHEJ, and consistent with a replicative origin
Source: Genome Res. 2019 May;29(5):737–49. doi: 10.1101/gr.240705.118 (PMC6499312; doi:10.1101/gr.240705.118)
Supplement: Supplemental Material [file supp_gr.240705.118_Supplemental_file_1.zip › contigs/annotated_contigs/DB84/contig.2.DB84_length_721_mean_cov_6.86546463245.docx]

**DB84_length_721_mean_cov_6.86546463245**

```
TGGTAGATCTACTTTTAGTTCTTTAAGGAATCTCCATACTGTTTTCCATAGTGGTTGTACTAGTTTACATTCCCACCAGCAGTATGTGA
 >chrX:95966214-95966538 + E=4e-183
AAGTGTTCCCTTTTCACCACATCCATGCCAACATCTATTATTTTTTGATTTTTAGATTATGGCCATTCTTGCAGGAGTAAGGTGGTATC

GCATTGTGGGTTTAATTTGCACTTCCTTGATAATTAGTGATGTTGAGCATTTTTTCATGTTTGTTGGCCATTTGCATATCTTCTTTTGA

GAATTGTCTATTTATGTCCTTTGCCCACTTTTTGATGGGATTATTTGTTTTTTTCT|T|TTCAACTTTTGTTTTAGATCGGGGTACATA
                                                         >chrX:95773189-95773587 + E=1e
GGCAGGTTTGTTACATGGGTATATCACACGATGCTGAGGTTTGGGGTACAGTTGAGCCCGTCACCCAGAAAATGAGCATAGTACCCAAA
-227
AGTTAGTTTTTCAACCACTACTCACCTCCCTCCGTTCCCCCTCTAGTAGTCCCCAGTGTCTATTGTTGTCATCTTTATGTCCACGAGTA

CCTAATGTTTAGTTCCCACTTATAAGTGAGAACATTCAGTATTTGGCTTTCTGTTTCTGCATTAATTTTCTTAGGATAATAGCCTCCAG

CTGTATCCATGTTGCTGCAAAGGACATAATTTCATTCTTTTTTATGGCTGCACAGATTCCATGTTGTATATGTACCACCTTTTCTTTTT

CCAAGCCACCA
```
